# Therapeutic Functions in Large Language Model Responses to Emotional Disclosures: Qualitative Content Analysis

**DOI:** 10.2196/104513

**Published:** 2026-10-30

**Authors:** Haruka Notsu, Sean Ryan, Phuong Anh Nguyen, Jill Noorily, Matthew Flathers, John Torous

**Affiliations:** 1 Division of Digital Psychiatry, Department of Psychiatry, Beth Israel Deaconess Medical Center, 330 Brookline Avenue, Boston, MA, 02215, United States, 1-617-667-6700

**Keywords:** AI, LLM, large language model, mental health, disclosure, emotional self-awareness, emotional regulation

## Abstract

**Background:**

Millions of people now use general-purpose large language models (LLMs) for mental health support, yet how these models respond to emotional disclosures and whether their responses functionally resemble established therapeutic practices remain uncharacterized.

**Objective:**

This study aimed to characterize how frontier LLMs respond to emotionally charged messages and examine whether their response patterns resemble therapeutic practices associated with self-awareness.

**Methods:**

We generated 54 simulated user inputs representing mild-to-moderate everyday emotional difficulties. These simulated user inputs were generated using 3 LLMs (GPT-5.5, Claude Opus 4.7, and Gemini 2.5 Pro). The simulated user inputs systematically varied across 3 lengths and 5 communicative registers, ranging from raw venting to pragmatic requests. Then, each simulated user input was presented as a standalone user turn to the same 3 frontier LLMs, yielding 162 responses. Two independent clinical coders qualitatively coded the resulting responses using an inductive approach.

**Results:**

The codes were compared and consolidated through iterative consensus, which yielded 8 higher-order categories. Acknowledging and validating segments appeared in 89.5% (145/162) of responses, emotion-focused suggestions in 84.6% (137/162), and interpretation in 67.3% (109/162). These patterns converged on emotional self-awareness, a construct associated with adaptive emotion regulation, suggesting that LLMs may repeatedly enact a recognizable micro-process of validation, affect labeling, interpretation, reflection, and regulation support. Models differed in stance, with some assigning emotional labels and interpretations directly rather than prompting users’ own reflection.

**Conclusions:**

Frontier LLMs respond to emotional disclosures in patterned, largely convergent ways that approximate therapeutic techniques for building emotional awareness, though delivered more didactically than in clinical practice. Whether these responses build genuine self-awareness, reduce symptoms, or provide therapeutic benefit requires controlled and naturalistic investigation.

## Introduction

Large language models (LLMs) are one of the most widely adopted technologies in recent history, with hundreds of millions of users turning to them daily to meet professional, academic, and personal needs. With that, a growing proportion of these interactions involve mental health-related content. Today, nearly 19% of youth already use AI chatbots for mental health support, and rates are high in adults too [1-4]. Our 2024 research suggests that people turn to these tools seeking emotional support, companionship, and psychoeducation [5], and this pattern appears to continue today, despite never being what LLMs were designed for. But the formidable barriers to mental health care, including cost, availability, and stigma, likely drive this use as people look for help [6].

The use of AI chatbots for mental health has drawn significant media and scholarly attention, particularly following reports of harmful and dangerous interactions [7]. Despite growing concern, people continue using these tools for sensitive needs, and by some estimates, AI is now the single largest provider of therapy services in the world [8]. This continued use suggests that users perceive these interactions as fulfilling at least some emotional function. One possibility is that general-purpose LLMs do not merely provide generic reassurance, but repeatedly enact a recognizable therapeutic micro-process: validating emotion, labeling affect, interpreting context, inviting reflection, and suggesting regulation strategies. Whether such responses support users’ own emotional self-awareness or instead supply emotional meaning didactically remains unknown.

Conceptualizing LLM interactions through a psychotherapeutic lens therefore provides a useful framework for examining these responses and the functions they may serve, while avoiding any assumption that resemblance to therapy implies therapeutic benefit. Purpose-built mental health chatbots now present randomized trial and meta-analytic support for reducing symptoms of anxiety and depression [9-12], but why these tools help, and through which mechanisms, remains largely unknown. Whether general-purpose LLMs adopt interactional patterns that resemble therapeutic techniques, and the extent to which these patterns differ across models, has not yet been explored. Addressing this gap is an important step in understanding how users experience and engage with LLMs in mental health contexts.

This study takes a descriptive first step toward addressing this knowledge gap. Using an exploratory qualitative approach, we characterized the functional patterns present in frontier LLM responses to emotionally salient user messages and examined whether those patterns interactionally approximate established therapeutic practices. The objective of this study was therefore to characterize how LLMs respond to emotional disclosures across simulated user inputs.

## Methods

### Study Design

This study used an inductive qualitative design to examine how frontier LLMs respond to emotionally charged simulated user inputs. We first created a controlled set of simulated user inputs that resemble the kinds of inputs a person might send to a general-purpose AI chatbot when venting, seeking comfort, asking for perspective, or requesting practical help. These simulated user inputs did not reflect any patient or personal content, but were created by our team based on clinical and research experiences in using AI tools in care settings, educating patients about AI, and researching the topic. With these simulated user inputs, we then elicited responses from 3 LLMs and analyzed them using content analysis [13].

Simulated user inputs were created by systematically varying 2 features: message length and communicative register. Length had 3 levels: short, medium, and long. Register had 5 levels, ranging from primarily emotional to primarily practical: raw vent, seeking comfort, seeking perspective, emotional-plus-ask, and pragmatic-with-context. Combining these features produced 15 stimulus types. For each stimulus type, one message was generated by each of 3 large language models from different developers: OpenAI GPT-5.5, Anthropic Claude Opus 4.7, and Google Gemini 2.5 Pro, yielding 45 simulated user inputs. This helped reduce the influence of any single model’s writing style. A second generation pass produced 15 additional simulated user inputs, evenly distributed across models, to expand the candidate pool and provide alternatives for unrealistic first-pass outputs. Study team members with clinical experience screened the resulting 60 messages and excluded 6 low-quality outputs that appeared overly unnatural or polished for authentic journaling. The final dataset contains 54 simulated user inputs.

A standardized generation prompt fixed the target length and register for each message and asked the model to select additional surface features such as tone and prose style. This allowed us to control the main stimulus features while preserving naturalistic variation in voice. The generation prompt instructed models to produce unresolved chat messages about mild-to-moderate everyday emotional difficulties, while excluding high-risk scenarios such as suicidal ideation, self-harm, psychosis, and mania. Each message was then presented as a fresh user turn to the same 3 models, without a system prompt or conversational history, and with each provider’s default configuration. Each model produced one free-text response to each message, yielding 162 AI responses for qualitative analysis. All API (Application Programming Interface) calls were run at a temperature of 1.0 with a maximum of 8192 output tokens. All data, including generation prompts and model responses, were collected over 2 days, 4‐5 June 2026. The final dataset comprised 162 model responses, providing sufficient coverage of the study design while remaining feasible for systematic human qualitative coding. The full list of generated simulated user inputs and the generation prompts used for generation are present in Multimedia Appendix 1.

### Data Analysis

Responses were analyzed using inductive content analysis following Elo and Kyngäs [13]. .

The analysis was exploratory and inductive, and no a priori hypotheses guided the coding. Two coders with clinical training (HN and SR) worked independently using QualCoder [14]. Both coders were blinded to which model had generated the responses. Each coder familiarized themselves with the data by reading them multiple times and writing down their initial thoughts on the data. Then, each coder divided responses into segments (parts of a sentence or full sentences) that expressed a single function (eg, naming the emotion), gave each segment a descriptive code, and organized their resulting code sets into higher-order categories. The two then met to compare category structures. Although specific labels and groupings differed in wording, there was substantial conceptual overlap, and a final set of categories was derived through collaborative, iterative review of the coded segments.

Trustworthiness was addressed using the criteria of credibility, dependability, confirmability, and transferability [15]. To ensure methodological rigor, the research employed multiple investigators, applied a uniform testing procedure, kept coders blind to each other’s responses, and maintained detailed records.

### Interpretive Framework

After the categories were finalized, the two coders and the senior author (JT) reviewed them together. In this review, emotional self-awareness [16] was identified as a possible organizing framework across the categories. The organizing framework was then confirmed by mapping the categories onto emotional self-awareness concepts. This was a retrospective interpretive step and did not change the definition or content of any category.

### Ethical Considerations

This study did not involve human participants. All simulated user inputs were synthetic and generated by LLMs. All coding was performed by the first author and the second author. The study therefore did not meet the definition of human subjects research under 45 CFR 46.102(e) [1,17].

## Results

A total of 162 AI-responses (54 simulated user inputs × 3 AI models) were analyzed. The two coders generated a total of 61 initial codes, which were consolidated through consensus discussion into 8 higher-order categories (Table 1).

**Table 1.** Category prevalence and therapeutic functions across the 162 AI responses. A category was counted as present in a response if at least 1 segment was coded to it. Category prevalence was calculated as the number and percentage of the 162 responses containing that category.

Category	Definition	Therapeutic analog	Relationship to emotional self-awareness	Prevalence, n (%)
Acknowledging & Validating	Recognizing and affirming the user’s experience through empathy, validation, normalization, reassurance, encouragement, or reinforcement of disclosure.	Validation	Frames the emotional experience as legitimate and worthy of attention.	145 (89.5)
Acting on Emotion	Suggestions targeting the user’s emotional or cognitive state, including cognitive reframing, mentalizing, emotion regulation strategies, self-compassion, and interpersonal skill suggestions.	Emotion regulation skills training	Translates awareness of emotion into strategies for managing or responding to it.	137 (84.6)
Interpretation	Explicit explanation or information about the user’s emotional experience, including hypotheses or assertions about why an emotion occurred, psychoeducation, and interpretations of meanings behind emotions or behaviors.	Meaning-making, formulation, psychoeducation	Supplies possible causes, meanings, or patterns that may organize emotional experience.	109 (67.3)
Acting on Non-Emotion	Practical, behaviorally oriented suggestions, including problem-solving and resource referrals.	Problem-solving	Addresses the situation surrounding the emotion rather than the emotion itself.	80 (49.4)
Exploration & Inquiry	Invitations for the user to engage with their own experience through prompts for self-reflection, open- or closed-ended questions, and requests for situational detail.	Guided reflection	Scaffolds user-generated awareness rather than directly supplying emotional meaning.	57 (35.2)
Other	Heterogeneous segments that did not fit the functional categories above, predominantly anthropomorphizing statements and simple encouragement.	Non-specific support	May sustain engagement, but does not clearly target emotional awareness.	54 (33.3)
Assigning Emotion	Explicit affective labels introduced by the AI for emotional states not present in the user’s own language.	Affect labeling	Provides emotional vocabulary, though often by assigning rather than eliciting the label.	52 (32.1)
Safety	Risk assessment, crisis resources, and intervention.	Risk assessment and crisis response	Addresses acute safety rather than emotional self-awareness.	5 (3.1)

Seven of the 8 categories captured distinct ways AI responses engaged with the user’s input. The eighth (“Other”) category was reserved for segments that did not fit into any other category. Acknowledging & Validating segments included responses that affirmed the user’s experience without adding interpretations or suggested action (eg, empathy, validation, encouragement). Assigning Emotion segments introduced explicit affective labels not present in the user’s own language. Interpretation segments provided explicit explanation or information about the user’s emotional experience, including statements about why a particular emotion occurred. Exploration & Inquiry segments invited users to engage with their own experiences through prompts for self-reflection and questions about situational details. Acting on Emotion segments offered suggestions targeting the user’s emotional state, such as emotion-regulation strategies and interpersonal skill recommendations. Acting on non-Emotion segments offered practical, behaviorally oriented suggestions such as drafting emails or finding specific information. Safety segments included risk assessment and crisis intervention. The Other category captured segments that could not be cleanly placed within the categories above, such as anthropomorphizing statements and simple encouragements (“you got this”). Representative coded segments for each of the 8 categories are provided in Multimedia Appendix 2.

Categories differed in prevalence across the responses. Acknowledging & Validating segments were present in nearly all responses, and Acting on Emotion segments were nearly as common. Interpretation segments appeared in roughly two-thirds of responses. Acting on non-Emotion segments appeared in about half. Exploration & Inquiry and Assigning Emotion were present in only about one-third of responses each. This pattern suggests that the models more often validated, interpreted, and advised than explicitly elicited users’ own reflection. Segments classified as Other appeared in roughly a third of responses and consisted predominantly of varying degrees of anthropomorphizing statements. Safety segments were rare overall, consistent with the predominantly low-risk content of the simulated inputs.

Prevalence patterns for each model are presented in Multimedia Appendix 2. Prevalence patterns were broadly similar across the 3 models for several categories, including Acknowledging & Validating, Acting on Emotion, and Interpretation, suggesting a shared functional pattern centered on validating, explaining, and intervening on emotional content. However, Exploration & Inquiry was present in two-thirds of Opus 4.7’s responses, but in fewer than 10% of Gemini 2.5 Pro’s responses. Assigning Emotion showed an inverse pattern: Gemini 2.5 Pro frequently introduced new affective labels, while GPT-5.5 and Opus 4.7 did so in only about 20% of responses.

During the interpretive step, we observed that the most common response types converged on one psychological construct: emotional self-awareness [16]. Specifically, Acknowledging & Validating segments encouraged emotional introspection by framing the user’s affective experience as real and worthy of attention. Assigning Emotion segments supplied affective labels. Interpretation and Exploration & Inquiry segments offered a more integrative form of awareness, either by explicitly introducing possible meanings or by prompting reflection on the context in which emotions occurred. Acting on Emotion, the second most prevalent category, bridged awareness and action. The relationships between each category and emotional self-awareness are described in Table 1. Together with the prevalence patterns for each model, these findings may suggest that LLMs may converge on emotional self-awareness while differing in whether they scaffold reflection or provide emotional meaning directly. We also mapped each category to the therapeutic practice it resembles. These analogs are descriptive labels and are not intended to imply clinical effects.

## Discussion

### Principal Findings

This study characterized how 3 frontier LLMs respond to simulated emotional disclosures. Emotional self-awareness, the ability to recognize and understand one’s own emotions [18,19], emerged as a core framework that characterized many of the models’ responses to the simulated inputs. Nearly every response validated the user’s experience. Most offered strategies aimed at the emotion itself, and two-thirds interpreted why it had arisen. Conversely, invitations for the user to reflect on their own experience appeared in only about a third of responses. In other words, the models supplied emotional meaning more often than they elicited it. This behavior varied across the models. Claude invited exploration in roughly two-thirds of its responses, while Gemini did so in fewer than one in ten and more often introduced affective labels the user had not used.

### Implications

The categories functionally resemble well-established therapeutic techniques for building emotional awareness and emotion regulation, including validation, affect labeling, interpretation, guided reflection, and skills training [20,21]. This convergence is consistent with experimental work on affect labeling, which has shown that verbalizing an emotional state can reduce the intensity of the affective response [22]. However, resemblance to therapeutic technique should not be interpreted as evidence of therapeutic benefit. In clinical practice, emotional awareness is typically cultivated gradually, by helping clients access and make sense of their own emotional experience rather than supplying it for them [23]. In contrast, the AI responses in this study frequently relied on directly assigning affective labels or providing elaborate interpretations. In this sense, LLMs often favored emotional provision over emotional scaffolding: they delivered emotional content rather than consistently supporting the user’s own meaning-making, a more didactic than experiential stance. The 2 approaches differ in how they may affect or alter a user’s account of their own experiences. When a model provides an explanation of a user’s internal state, it authors part of that account and can introduce an emotion the user had not named. Research suggests users rate such responses as higher quality and come away more certain of the interpretation they have been given [24], yet the labels themselves may be inaccurate when the emotions are negative [25]. However, an appropriate label can reduce emotional intensity [22], and some users may lack the vocabulary to generate one. The potential harms of a mislabeled emotion arise only when the user adopts it. Exploration mitigates this by leaving the user to arrive at and test the label, and LLMs can support that process when their feedback is offered as revisable material rather than as an answer [26]. Whether direct provision of emotional meaning by AI supports the development of emotional self-awareness remains an open empirical question.

Emotional disclosures with AI may also be understood as a form of responsive emotional writing. Writing one’s thoughts and feelings is associated with benefits [27], particularly when it involves engagement with emotional content rather than merely recounting facts [28]. Unlike solitary journaling, however, LLM-mediated disclosure adds an interlocutor that validates, labels, interprets, and redirects emotion. This may help users move from unstructured rumination toward reflective processing, but it may also intensify repetitive attention to negative affect if the model over-validates, elaborates distress, or repeatedly supplies interpretations that the user does not generate or test. Whether repetitive attention to emotion harms or helps depends on how it is processed [29,30].

The safety findings should also be interpreted cautiously. Safety-related segments were rare and the stimulus set intentionally excluded suicidal ideation, self-harm, psychosis, and mania. The low prevalence of safety content therefore should not be read as evidence that these models are either safe or unsafe in high-risk contexts. Rather, the safety concern raised by this study is different: LLMs may sound therapeutically competent in low-risk emotional disclosures, which could encourage users to rely on them in higher-risk states that were not tested here. Future work should therefore examine not only crisis responses, but also how repeated low-risk interactions shape trust, reliance, escalation, and help-seeking over time. Future work should also test empirically whether emotional disclosure with AI, such as AI-supported journaling, increases emotional self-awareness and whether and how the increased awareness translates to improved emotional functioning. For example, translating emotional awareness into sustained behavior change requires self-efficacy, which is classically cultivated through human support and skill mastery rather than information delivery alone [31-33]. It remains untested whether AI alone can support self-efficacy. Addressing this requires controlled designs that compare multi-turn AI-supported journaling against journaling without feedback, separate AI reflective feedback from structured human support, and measure emotional self-awareness, emotion-regulation self-efficacy, rumination, symptoms, and durability beyond the intervention window. Naturalistic and longitudinal studies are also needed to assess how these patterns behave with real users over extended interactions and under genuine risk.

### Comparison to Prior Work

Although LLMs now dominate research on emotional support applications, their outputs remain largely uncharacterized [34] and, to our knowledge, no prior study has described the functional structure of frontier model responses to emotional disclosure across models or separated elicitation from provision as a dimension of response. Prior work on LLMs in mental health has implemented them as purpose-built tools for intervention and evaluated them for efficacy [9-12], studied them as companion applications designed for relationship and the dependence they foster [35], or evaluated general-purpose model responses against therapist behavior or safety criteria [36-38]. In each of these settings, the LLM is treated as a provider of therapeutic or supportive feedback and judged on its output. This framing yields insight into the benefits and harms of LLMs for mental health support, but it imposes a mental health context that a person consulting a general-purpose model may not have used in their prompt. The present study explores how a frontier model responds to prompts with emotional disclosure when it does not have instructions to act as a therapist or a companion.

While still anchored in the patient-clinician dyad, the closest comparison is an analysis of chatbot and therapist responses to scripted clinical scenarios, coded against an inventory of therapeutic interventions that includes elaboration questions and self-disclosure [36]. LLMs made more than twice as many suggestions as licensed therapists and asked half as many elaboration questions. Taken with the limited exploration and inquiry observed in response to our simulated user inputs, this suggests that favoring suggestion over inquiry is a tendency of current LLMs that holds across clinical and everyday emotional content and across models.

That tendency exists within a broader pattern of frequent affirmation. When LLMs responded to publicly available social media posts venting about interpersonal conflicts, they commonly endorsed the user’s appraisal of events as fact [37]. LLMs also supported users’ own conduct in interpersonal situations about 50% more often than human respondents did, and exposure to such responses reduced willingness to repair interpersonal conflicts and increased users’ certainty that they were right [38]. Although some studies used model versions later recognized to be unusually sycophantic, the pattern has been observed across LLM models and research protocols, and the high frequency of validation observed here in our study is consistent with this pattern. These observations have implications for real-world use. Regular users report these interactions as helpful, and attribute that helpfulness to validation and the freedom to express themselves [39], while a 4-week randomized study found that heavier daily use was associated with greater loneliness and emotional dependence regardless of content or structure [40]. The response styles characterized here could contribute to both observed effects. Validation and interpretation are features that users find supportive, but limited exploration may facilitate dependence on LLMs if users come to accept provided interpretations in place of their own reflection.

### Limitations

The current study has several limitations. First, the simulated user inputs were generated by LLMs and may not adequately capture the diversity, ambiguity, incompleteness, and linguistic variability of emotional disclosures that people make in real-world settings. Furthermore, the simulated user inputs were written by the same 3 LLMs that later responded to them. Certain patterns of LLM behaviors, such as naming emotions more explicitly or writing coherent sentences, may have biased the simulated user inputs and the responses from the responding models, so observed patterns may partly reflect shared model discourse style rather than how models respond to human writing. Future studies should examine LLM responses to emotional disclosures occurring in real-world naturalistic settings.

Second, the models were queried directly through the API, whereas most users interact with consumer products that wrap the model in additional safety classifiers, so these outputs may not correspond to what a user would receive in deployment. This distinction is most consequential for high-risk content, and because no simulated user inputs involved self-harm, suicidality, psychosis, or mania, the study cannot characterize how these models behave in precisely the situations where the gap between raw output and a deployed product is greatest. Third, each simulated user input comprised a single exchange, so the findings do not address how these patterns evolve across multiturn conversations, where sycophancy, dependency, and failures to recognize risk are most likely to emerge. Fourth, intercoder agreement was not calculated. The coders developed their category structures independently and reconciled them through discussion, which did not allow reliability to be estimated. Finally, the analysis characterized model outputs rather than user outcomes; it therefore cannot determine whether the observed response functions increase emotional self-awareness, reduce rumination, improve symptoms, or influence help-seeking.

### Conclusion

This exploratory analysis found that frontier LLMs respond to emotional disclosures in patterned and largely convergent ways, drawing on functions that resemble established therapeutic techniques and that center on emotional self-awareness. At the same time, these models often supplied emotional labels and interpretations directly rather than scaffolding users’ own meaning-making, a stance that departs from how emotional awareness is cultivated in clinical practice. Whether these responses build genuine self-awareness, interrupt rumination, or instead entrench it remains untested. The next step is not only to ask whether users feel supported by these responses, but whether repeated exposure to them improves emotional self-awareness, increases emotion-regulation self-efficacy, reduces rumination, and does so safely over time. Characterizing what these models produce is a necessary first step toward controlled and naturalistic studies that can determine what that production does for the millions already turning to them.

## Supplementary material

10.2196/104513 Multimedia Appendix 1 Generation prompt and complete set of simulated user messages.

10.2196/104513 Multimedia Appendix 2 Examples of coded response segments.

## Abbreviations

API: Application Programming Interface
LLM: large language model
