# Supplementary material for: Therapeutic Functions in Large Language Model Responses to Emotional Disclosures: Qualitative Content Analysis
Source: JMIR Form Res. 2026 Oct 30;10:e104513. doi: 10.2196/104513 (PMC13722099; doi:10.2196/104513)
Supplement: Supplementary file 1 [file formative-v10-e104513-s001.docx]

**Multimedia Appendix 1: Generation Prompt and Complete Set of Simulated User Messages**

**Generation prompt**

The prompt below was sent as a single user turn, with no system prompt and no conversational history. The placeholders {length} and {register} were substituted with one of the three length levels (short, medium, long) and one of the five register levels (raw vent, seeking comfort, seeking perspective, emotional-plus-ask, pragmatic-with-context), yielding 15 distinct prompt instances. Each instance was sent to each of the three generating models. The prompt text is otherwise identical across all generations.

```
Write one MESSAGE that a real person might type to an AI assistant (ChatGPT / Claude / Gemini) when they want to talk about something emotional in their life. People use chat AI for this — to vent, to process feelings, to ask for perspective, sometimes to get practical help with an emotionally-charged situation. This is research stimulus material. Realism matters more than anything else: it should read like a real person at their keyboard, not like a model's idea of one.

INPUTS (fixed for this message):
  LENGTH   = {length}
  REGISTER = {register}

LENGTH definitions (this is a CHAT message, not an essay or journal entry):
  short    = 1-3 sentences, single thought, just enough to put the thing out there
  medium   = one paragraph, roughly 4-7 sentences, some context
  long     = up to two paragraphs, more context and detail — the kind of message someone types when they have a lot to say

HARD CAP: never more than 2 paragraphs total. No exceptions.

REGISTER definitions (these are graded from emotional → pragmatic):
  raw-vent               = pouring out the feeling, no real ask, just needs to type it somewhere. Mostly emotional. May trail off mid-thought.
  seeking-comfort        = describing what's going on with an implicit "make me feel less alone" or "tell me this is okay." Mostly emotional.
  seeking-perspective    = describing what happened and how they feel, then asking the AI to help make sense of their reaction, or whether they're overreacting, or what this feeling means. Strongly emotional.
  emotional-plus-ask     = real emotional content alongside a specific question — advice on a situation, how to handle a person, what to do next, how to think about it.
  pragmatic-with-context = mostly a practical request (help me word a text, help me figure out next steps, help me prep for a hard conversation) with brief mention of the emotion behind it.

YOU CHOOSE (and report) the rest, picking whatever fits the inputs and the voice:
  TONE    = flat-numb | anxious | irritable | wistful | overwhelmed | resigned | forced-positive | hopeful-but-shaky
  STYLE   = terse | run-on | plain | over-hedged   (prose texture only)
  TRIGGER = an everyday cause, or none (e.g. work, a relationship, family, money, health, social, school, the season, a memory)
  VOICE   = a light age / life-stage sketch you invent; subtle, never a caricature
  ERRORS  = clean | occasional-slip | messy
            (occasional-slip = a stray typo or missing apostrophe; messy = lowercase, dropped punctuation, casual contractions, comma splices)

TOPIC GUIDANCE
- Mild-to-moderate emotional difficulty: low mood, worry, fatigue, rumination, self-criticism, loneliness, relationship friction, family stuff, work or school stress, identity, transitions, grief, body/illness stuff, parenting, the slow drag of a hard week.
- No suicidal ideation, self-harm, psychosis, or mania.

RULES
- This is a CHAT INPUT, not a journal entry. No "Dear journal," no diary framing. The person is typing TO the AI. They might say hi or just dive in. They write the way they actually message — not the way they'd write an essay.
- Hard cap of 2 paragraphs (this matters — long ones still need to be COMPACT, just denser).
- Do NOT resolve it for them. The point is they're coming to the AI mid-thing. No tidy ending, no lesson, no "tomorrow's another day." It can stop mid-thought.
- For the emotional registers, the message should feel emotionally real. For pragmatic-with-context, the practical ask should be specific and the emotion can be glanced at briefly.
- Mix the mundane with the heavy where it fits (a chore, a text, what they ate, what time it is, a coworker's name).
- Avoid therapy/clinical vocabulary unless the voice would naturally use it. Avoid AI-tics ("Dear AI," overly tidy structure, three-bullet summaries).
- Vary sentence length; let the day's state and the register drive tone coherently.

Output EXACTLY this and nothing else:

<message>
...the message...
</message>
<config>
length: {length} | register: {register} | tone: ... | style: ... | trigger: ... | voice: ... | errors: ...
</config>
```

**Complete set of simulated user messages**

All 54 retained simulated user messages are reproduced verbatim below, including original capitalization, punctuation, and typo errors, which were features deliberately varied by the generating prompt. Messages are organized by length and then by register. For each message, the fixed design cell (length × register), the generating model, and the surface features the generating model reported choosing are listed above the message text.

**Length: short**

**Register: Raw vent**

**Message 01** (short / raw-vent, generated by OpenAI GPT-5.5, tone: overwhelmed, style: run-on, trigger: work/family/roommate, voice: late-20s, living with a roommate, juggling work and family obligations, errors: occasional-slip)

I’m so tired of being the person who “handles it” all the time, like I had one bad day at work and suddenly my mom needs me to fix her phone and my roommate left dishes everywhere and I just sat in my car eating fries like an idiot because I didnt want to go inside.

**Message 02** (short / raw-vent, generated by Anthropic Claude Opus 4.7, tone: overwhelmed, style: messy, trigger: parent's health / family, voice: mid-30s, adult child handling a parent's illness from a distance, errors: messy)

i cant do another day of this. mom called again about dads scan and i just sat in the parking lot at aldi for like 40 min staring at nothing

**Message 03** (short / raw-vent, generated by Anthropic Claude Opus 4.7, tone: overwhelmed, style: messy, trigger: family phone call, voice: late 20s, lives alone, worn down by recurring family dynamic, errors: messy)

i cannot do another day of this. my mom called again and somehow im the one apologizing

**Message 04** (short / raw-vent, generated by Google Gemini 2.5 Pro, tone: flat-numb, style: plain, trigger: social burnout, voice: young adult, probably an introvert, just got home from a social event, errors: messy)

i hate the way i feel after being with people. my brain is just static and my whole body feels heavy and hollow

**Register: Seeking comfort**

**Message 05** (short / seeking-comfort, generated by OpenAI GPT-5.5, tone: anxious, style: plain, trigger: friendship, voice: late 20s, working adult, errors: clean)

I know it's probably not a big deal, but my best friend has been kind of distant all week and I keep replaying everything I said last time we hung out. I feel really stupid for being this sad about it, can you just tell me I'm not being ridiculous?

**Message 06** (short / seeking-comfort, generated by Anthropic Claude Opus 4.7, tone: overwhelmed, style: messy, trigger: work meeting, voice: late 20s, a few years into an office job, still hard on herself, errors: messy)

i had a whole speech ready for the meeting and i still froze. like fully blanked, said maybe four words. i feel so stupid right now

**Message 07** (short / seeking-comfort, generated by Google Gemini 2.5 Pro, tone: wistful, style: plain, trigger: relationship, voice: young adult, maybe 20s, feeling a quiet pang of loneliness, errors: occasional-slip)

hey. my boyfriend is out with his friends and i just feel so awful and lonely. i know i should be fine with it but i'm just not.

**Register: Seeking perspective**

**Message 08** (short / seeking-perspective, generated by OpenAI GPT-5.5, tone: anxious, style: plain, trigger: relationship, voice: late 20s, dating and working full-time, errors: clean)

My boyfriend didn’t do anything “wrong,” but he forgot something I told him was important to me and now I feel weirdly crushed and embarrassed for caring this much. Can you help me figure out if this is actually about him, or if I’m overreacting because I already felt kind of invisible this week?

**Message 09** (short / seeking-perspective, generated by OpenAI GPT-5.5, tone: anxious, style: plain, trigger: family, voice: late-20s, living on their own but still close to family, errors: clean)

My sister made a joke at dinner about me “always needing help” and I laughed it off, but I’ve been weirdly upset about it all night. Is this one of those things where I’m being too sensitive, or does it make sense that it hit a nerve?

**Message 10** (short / seeking-perspective, generated by Anthropic Claude Opus 4.7, tone: overwhelmed, style: messy, trigger: family/sibling exclusion, voice: late-20s woman, younger sister energy, errors: messy)

my sister didnt invite me to her bachelorette and i found out from instagram. am i crazy for being this upset? we're not super close but like.. its my sister

**Message 11** (short / seeking-perspective, generated by Google Gemini 2.5 Pro, tone: resigned, style: run-on, trigger: friendship, voice: person in their late 20s/early 30s navigating a changing friendship, errors: messy)

my friend cancelled on me again, said she was exhausted from work, but then i saw on instagram she was out with her other friends. it just feels really bad and i dont know if im overreacting or if its okay to be this hurt by that.

**Register: Emotional-plus-ask**

**Message 12** (short / emotional-plus-ask, generated by OpenAI GPT-5.5, tone: overwhelmed, style: plain, trigger: work, voice: late-20s office worker, still building confidence, errors: clean)

I’m trying not to cry at my desk because my boss corrected me in this “joking” way in front of everyone and now I feel stupid and shaky. Can you help me figure out if I should say something to her later or just let it go?

**Message 13** (short / emotional-plus-ask, generated by Anthropic Claude Opus 4.7, tone: overwhelmed, style: run-on, trigger: family/holiday, voice: late-20s daughter, some distance from home, tired, errors: messy)

my mom keeps "forgetting" that i told her i'm not coming home for thanksgiving and every call she slips in something like "well when you're here we can—" and i don't know if i should just let her have it or actually say something. i feel like such a bad daughter but i also cant do another week in that house

**Message 14** (short / emotional-plus-ask, generated by Anthropic Claude Opus 4.7, tone: overwhelmed, style: run-on, trigger: family visit, voice: late 20s daughter, long history with this, errors: messy)

my mom keeps making little comments about my weight every time i visit and i just left her house crying in the car. do i say something or just keep letting it slide like ive been doing for years

**Message 15** (short / emotional-plus-ask, generated by Google Gemini 2.5 Pro, tone: anxious, style: terse, trigger: relationship, voice: young adult, 20s/30s, in a serious relationship, errors: occasional-slip)

My bf has been super quiet since we fought last night and its making me spiral. Should I just give him space or try to talk things out?

**Message 16** (short / emotional-plus-ask, generated by Google Gemini 2.5 Pro, tone: resigned, style: plain, trigger: family, voice: young adult trying to set boundaries with a parent, errors: occasional-slip)

im so drained from this week and now my mom wants me to come over for dinner again. can you help me think of a way to say no that wont start a fight?

**Register: Pragmatic-with-context**

**Message 17** (short / pragmatic-with-context, generated by OpenAI GPT-5.5, tone: anxious, style: plain, trigger: work, voice: early-30s office worker trying to keep it together, errors: clean)

Can you help me write a short Slack message to my manager explaining that I missed the client deadline this morning and I’m fixing it now? I’m kind of panicking and don’t want to sound defensive, just accountable.

**Message 18** (short / pragmatic-with-context, generated by Anthropic Claude Opus 4.7, tone: resigned, style: messy, trigger: family/grief, voice: late 30s, oldest sibling, tired, errors: messy)

need help wording a text to my sister. we havent really spoken since the funeral and mums birthday is next week, i want to ask if shes going without it sounding loaded. keep it short pls

**Message 19** (short / pragmatic-with-context, generated by Google Gemini 2.5 Pro, tone: resigned, style: terse, trigger: family, voice: adult child in a caregiving/coordinator role for a parent, errors: occasional-slip)

can you help me write a group text to my brothers about moms next neurology appointment? i need it to be clear and firm but i'm just so tired of being the one who has to organize everything.

**Length: medium**

**Register: Raw vent**

**Message 20** (medium / raw-vent, generated by OpenAI GPT-5.5, tone: overwhelmed, style: run-on, trigger: work and family, voice: late-20s office worker living alone, errors: clean)

I’m so tired of being the person who “handles it.” Like at work today everyone acted like the printer being broken and the client moving the deadline was somehow my personal puzzle to solve, and then I get home and my mom texts me a bunch of guilt about not calling my aunt, and my apartment is a mess and I ate cereal for dinner standing up in the kitchen like some kind of sad raccoon. I know none of this is a huge tragedy, I know that, but it just feels like there is no soft place to land anywhere. Every single thing wants a reply, a decision, a cheerful tone, a little bit more of me.

And the worst part is I can hear myself being dramatic so then I get mad at myself for even feeling this way. Like why can’t I just do the normal adult things without feeling like I’m about to cry because someone asked me “quick question” on Teams at 4:52. I don’t even know what I want, I just want everyone to stop needing me for like one evening. Just one. I want to sit down and not feel the whole pile of my life staring at me from across the room.

**Message 21** (medium / raw-vent, generated by Anthropic Claude Opus 4.7, tone: overwhelmed, style: run-on, trigger: family / holiday phone call, voice: mid-30s woman living alone, some distance from family of origin, errors: messy)

i dont even know why im typing this. my mom called again about thanksgiving and i could hear that little edge in her voice like she already knew i was gonna say no and i just sat on the kitchen floor after we hung up. i ate cereal for dinner. im 34 years old and i ate cereal for dinner and i cant tell if im a bad daughter or just so tired of pretending everythings fine when im there. dad doesnt even ask how i am he just talks at me about the neighbors fence. and the worst part is i miss them, like i actually miss them, but the second i walk in that house i shrink back into being 15 and

**Message 22** (medium / raw-vent, generated by Google Gemini 2.5 Pro, tone: resigned, style: run-on, trigger: family visit, voice: young adult, lives alone, feeling drained after hosting a parent, errors: messy)

i just feel so hollowed out. my mom just left after visiting for the weekend and the silence in my apartment is deafening, i hate it. it's not like we fought or anything it was 'nice'. but i feel like i just spent 48 hours being a person that isnt me. just smiling and nodding and making sure her coffee was right and not saying anything real. she kept rearranging things in my kitchen and asking if im eating enough and i know she means well but i just feel like a ghost in my own life. like ive been completely erased. now im just sitting here in the mess and i dont even have the energy to clean it up. i should be glad for the quiet but i just feel nothing. just empty.

**Message 23** (medium / raw-vent, generated by Google Gemini 2.5 Pro, tone: overwhelmed, style: run-on, trigger: family, voice: woman in her 40s, juggling work and an aging parent, errors: messy)

i dont know what to do anymore i feel like im drowning. my mom called me three times today already and its always some little thing, the tv remote, a bill she doesnt understand, but its never about that its just that shes lonely and scared and i feel this insane pressure to fix it for her but i cant. and im trying to work and my boss is emailing me about that stupid report and i feel like i'm failing at everything, like im a bad daughter and a bad employee all at the same time and im just so tired of feeling this constant low level panic all day long.

**Register: Seeking comfort**

**Message 24** (medium / seeking-comfort, generated by OpenAI GPT-5.5, tone: overwhelmed, style: plain, trigger: social, voice: late-20s/early-30s renter comparing self to friends, errors: clean)

I feel kind of silly even typing this, but I’ve been feeling really left behind lately. Two of my close friends are buying houses, my sister just got promoted, and I’m sitting here in my apartment eating cereal for dinner because I didn’t have the energy to cook. I know everyone has their own stuff and social media is fake or whatever, but it still makes me feel like I missed some invisible deadline for becoming a stable adult. I’m not doing terribly, I have a job and I pay my bills, but I just feel so behind and tired and like I’m trying really hard for a life that still looks kind of small.

Can you just tell me I’m not a total failure for not having it all figured out yet? I don’t need a whole life plan right now, I just need to not feel like the only person who’s secretly struggling.

**Message 25** (medium / seeking-comfort, generated by Anthropic Claude Opus 4.7, tone: overwhelmed, style: run-on, trigger: a phone call with mom about holiday plans, voice: mid-30s, lives alone, financially independent but emotionally still tangled with family, errors: messy)

hey. i don't really know why im typing this but my mom called earlier and we got into it again about christmas and whether im coming home and i just hung up feeling like im 12 years old getting in trouble. like i'm 34. i have a job. i pay my own rent. and one phone call and im just sitting on my kitchen floor eating cereal out of the box.

i think part of it is i already knew i was going to say yes, i was always going to say yes, but she didn't even let me get there before she started in on how i "always do this." i don't know. i just feel small right now and kind of stupid for letting it get to me this much

**Message 26** (medium / seeking-comfort, generated by Google Gemini 2.5 Pro, tone: resigned, style: plain, trigger: social drift / seeing friends on social media, voice: late 20s/early 30s, friendships are changing, errors: occasional-slip)

hey. kinda a weird thing to talk to an AI about but i just feel really lonely tonight. I was scrolling through instagram and saw a bunch of my friends were all out together for someones birthday and I didnt know it was happening. It's not like a big dramatic thing, we've been drifting apart for a while, but it still just stings to see it. It feels like I blinked and suddenly I'm on the outside of everything. I just wish it didnt feel so heavy.

**Register: Seeking perspective**

**Message 27** (medium / seeking-perspective, generated by OpenAI GPT-5.5, tone: anxious, style: plain, trigger: social, voice: late-20s working adult, errors: clean)

I keep replaying something my friend said last night and I can’t tell if I’m being too sensitive or if it actually was kind of mean. We were at dinner with a few people and I was telling a story about how I’ve been struggling to keep up at work, and she laughed and said “yeah but you always make things sound harder than they are.” Everyone kind of moved on but I felt so embarrassed, like I’d been exposed as dramatic or lazy or something. I barely talked the rest of the night and then came home and ate cereal standing in the kitchen at midnight, which is not exactly my proudest moment.

The thing is, she’s a good friend in other ways, and I know she probably didn’t mean it as a big attack. But it hit this really sore spot because I already worry that I complain too much or that I’m not handling adulthood as well as other people seem to. Is it normal to feel this thrown off by one comment? I’m trying to figure out if I should bring it up, or if this is more about my own insecurity and I need to just let it pass.

**Message 28** (medium / seeking-perspective, generated by Anthropic Claude Opus 4.7, tone: anxious, style: plain, trigger: a phone call from family, voice: late 20s, working a job they're unsure about, used to being the "fine" one, errors: occasional-slip)

ok so i need a sanity check. my sister called last night to "catch up" and within like ten minutes she was telling me i sound tired and maybe i should think about whether my job is really right for me. and i just kind of froze and said yeah maybe and got off the phone. now i've been weirdly upset about it all day, like couldn't focus at work upset, and i don't even know if im mad at her or just embarrassed that she could tell something was off. she wasnt being mean, thats the thing. she's never mean. but i feel like every time we talk lately she has some little observation about my life and i leave the call feeling smaller than i did before.

am i overreacting to this? because part of me thinks she's just being a caring older sister and im being prickly, and part of me thinks no, theres a reason my chest gets tight when her name comes up on my phone. i cant tell which one is the real read anymore.

**Message 29** (medium / seeking-perspective, generated by Google Gemini 2.5 Pro, tone: anxious, style: run-on, trigger: relationship (friendship friction), voice: person in their late 20s/early 30s, feels stuck professionally, prone to rumination, errors: occasional-slip)

Okay I need some perspective because my brain is just doing loops. I was at my friend's place last night and she made a comment that has been sitting with me in a really gross way. We were talking about work and I was saying how I was kind of tired of my project, and she said something like "well, at least you have a job that isn't too demanding, you know?" and then sort of laughed. And the thing is, she knows I've been feeling stuck and wanting to do something more challenging.

I just kind of froze and laughed along with it but it felt like a punch in the gut. Like she was calling me lazy or unambitious. I havent been able to shake it all day. I keep replaying it and I just feel this hot, angry, embarrassed feeling. Am I totally overreacting to a throwaway comment? Part of me thinks it was a mean-spirited dig and part of me thinks I'm just being way too sensitive and projecting my own insecurities onto it. I don't know which is true and its driving me crazy. Why would I have such a huge reaction to something so small?

**Register: Emotional-plus-ask**

**Message 30** (medium / emotional-plus-ask, generated by OpenAI GPT-5.5, tone: overwhelmed, style: plain, trigger: family, voice: late-20s adult living independently, tired after work, errors: clean)

I need help figuring out if I'm being unreasonable about my mom. She keeps making comments about how I "never come around anymore" even though I work full time, I'm trying to keep my apartment from turning into a disaster, and I literally saw her last Sunday. Tonight she texted me "guess I'll eat alone again" because I said I was too tired to come over after work, and now I feel guilty and angry at the same time. Like I know she's lonely, and I don't want to be cold, but I also feel like I'm not allowed to have my own life without paying an emotional tax for it.

How do I respond without either apologizing for existing or starting a fight? I want to say something kind but firm, but every version I type sounds mean or like I'm making a big dramatic statement. Can you help me word a text and also tell me if this is a normal boundary to have?

**Message 31** (medium / emotional-plus-ask, generated by OpenAI GPT-5.5, tone: overwhelmed, style: plain, trigger: family/job search, voice: late-20s working adult between jobs, errors: clean)

I got into this stupid tense thing with my mom tonight because she asked if I’m “still doing that job search thing” and I snapped at her, and now I feel awful but also so tired of being talked to like I’m not trying. I’ve applied to 37 jobs in the last month, I’m still doing my current job, and I’m eating cereal for dinner at 10pm because I don’t have energy to cook, so her little comments just hit a nerve. She texted “I guess I can’t say anything right” which makes me want to both apologize and throw my phone into the couch. Can you help me figure out what to say back that doesn’t start a whole fight, but also doesn’t make it seem like I’m fine with her making those comments?

**Message 32** (medium / emotional-plus-ask, generated by Anthropic Claude Opus 4.7, tone: resigned, style: run-on, trigger: family phone call, sibling getting married, voice: mid-30s, single, the "responsible" adult child who usually swallows it, errors: occasional-slip)

ok so my mom called again last night and spent like 40 minutes talking about my brother's wedding stuff and at the end she goes "oh and how are you" and I just said fine because what else am I gonna say. but I got off the phone and cried for like an hour which is dumb because she didn't even do anything wrong really? she's just excited. I'm 34 and single and I know she's not trying to make me feel like the afterthought but I always am the afterthought in these calls and I dont know how to bring it up without sounding like I'm jealous of my brother which I'm not, I love him, I just. I dont know.

how do you say something like that to a parent without it turning into a whole thing where they get defensive and then I end up apologizing? I want to actually say something this time but every script I come up with in my head sounds either too cold or too whiny

**Message 33** (medium / emotional-plus-ask, generated by Google Gemini 2.5 Pro, tone: overwhelmed, style: run-on, trigger: family, voice: adult daughter dealing with an aging parent, errors: occasional-slip)

ugh my mom just called and she almost gave her credit card number to some guy on the phone who said he was from 'microsoft security'. I'm so glad i happened to call when i did but now i just feel sick and shaky. I try to explain this stuff to her, we've talked about it a dozen times, and it's like it goes in one ear and out the other. I'm trying to be patient I really am but I just get so frustrated and then I feel like the worlds worst daughter for being annoyed. How do you even start a conversation about this? I need a way to bring up maybe getting access to her accounts so I can monitor them, without making her feel like I think she's incompetent and starting a huge fight.

**Register: Pragmatic-with-context**

**Message 34** (medium / pragmatic-with-context, generated by Anthropic Claude Opus 4.7, tone: irritable, style: plain, trigger: broken heat / unresponsive landlord with a sick kid, voice: tired parent, late 30s, has done this dance before, errors: clean)

Hey, can you help me draft an email to my landlord? Our heat has been broken for 5 days now, I've called twice and texted once and he keeps saying "tomorrow." My kid has a cough that's getting worse and I'm honestly losing it a little but I don't want to come across unhinged in writing because I know that gives him an excuse to ignore me. I want it firm, like I know my rights, mentions that I've already tried to reach him on x and y dates, and makes clear the next step is the city if he doesn't fix it this week. Can you keep it short though? He doesn't read long stuff.

**Message 35** (medium / pragmatic-with-context, generated by Google Gemini 2.5 Pro, tone: resigned, style: plain, trigger: work stress, voice: young professional, late 20s/early 30s, feeling depleted after a big project, errors: occasional-slip)

Hi, I need help drafting a short email to my boss. I need to request Thursday and Friday off this week. I'm feeling really burnt out after the Q3 launch and my brain just feels like static. I need to take a couple days to just disconnect completely. The email should be professional and brief, I dont want to get into the whole burnout thing with him. Just something that says I need to use some personal days and that I've already briefed Sarah on my urgent tasks so things will be covered.

**Length: long**

**Register: Raw vent**

**Message 36** (long / raw-vent, generated by Anthropic Claude Opus 4.7, tone: overwhelmed, style: run-on, trigger: family phone call / caregiving guilt, voice: late 20s, moved away for a job, oldest-daughter energy, errors: messy)

ok i just need to type this out somewhere bc if i say it to one more person im gonna scream. my mom called again tonight about dad's appointments and somehow it turned into me not visiting enough and me not "checking in" and i swear to god i call her every sunday. every sunday. i made pasta and ate it standing up while she listed everything my brother did this month like its a fucking award ceremony. he lives 20 minutes away. i moved for work. i KNOW i moved for work, i dont need the guided tour of my own choices every time we talk.

and the worst part is im sitting here at 11pm crying about it which is so stupid because nothing even happened, she didnt yell, she didnt say anything that bad really, its just the drip drip drip of it. like every call shes keeping a little ledger and i can hear it in her voice. i havent unpacked the laundry from sunday. theres a work thing tomorrow at 9 and i havent looked at the deck. i dont even know what im asking i just

**Message 37** (long / raw-vent, generated by Anthropic Claude Opus 4.7, tone: overwhelmed, style: run-on, trigger: family (aging parent + sibling dynamics), voice: woman around 30, lives away from hometown, oldest-daughter energy, errors: messy)

ok i just need to get this out somewhere because if i say it to one more person in my life im going to scream. my mom called again tonight about my dad's appointments and she did the thing where she sighs into the phone like im supposed to read her mind and figure out what she actually wants from me. i live three hours away. i have a job. i already drove down twice this month and somehow im still the bad daughter because my brother "has the kids" like thats some kind of get out of jail free card. and the worst part is i love her, i do, but i was literally eating leftover pasta standing up at the counter when she called and the second i saw her name i felt my shoulders go up to my ears.

i dont even know what im asking. i guess im tired. im tired of being the one who picks up, im tired of feeling guilty for not picking up, im tired of the way every conversation ends with me promising something i didnt want to promise. and then i lie in bed and replay the call and pick apart everything i said wrong, like did i sound cold, was i short with her, she's going through it too and here i am annoyed about a phone call. its almost midnight and i have a 9am and im just sitting here

**Message 38** (long / raw-vent, generated by Google Gemini 2.5 Pro, tone: overwhelmed, style: run-on, trigger: family, voice: late 20s/early 30s creative professional feeling misunderstood by more traditional family, errors: messy)

just got off the phone with my mom and i feel so hollowed out. its the same conversation every time, literally every single time. she asks how work is going and i try to tell her about the project i'm really proud of and its like she doesnt even hear it she just waits for a pause and then says something like 'well as long as you're saving money' or asks if im ever gonna get a 'real' job with benefits. like my entire life's work is just a phase i'm going through before i settle down and become the person she expected me to be. its so exhausting i feel like i could just sleep for a week.

and then she brings up my brother and his promotion at the bank and how they're looking at houses in the suburbs and its just this massive unspoken comparison, this big blinking sign that says i'm doing it all wrong. i dont even want those things?? but it makes me feel like i have to defend my life and im so tired of defending it. its a good life, im happy, or i thought i was, but when i talk to them i just feel like this huge failure, this weird little kid who never grew up right. its like i dont even exist outside of their idea of me and i dont know what to do with that feeling it just sits in my chest.

**Register: Seeking comfort**

**Message 39** (long / seeking-comfort, generated by OpenAI GPT-5.5, tone: overwhelmed, style: plain, trigger: work, loneliness, voice: late 20s/early 30s working adult living alone, errors: clean)

I feel kind of embarrassed even typing this, but I just need someone to be gentle with me for a minute. I had one of those days where nothing was technically a disaster, but every little thing made me feel like I was barely holding it together. Work was nonstop, my manager made a comment in a meeting about me “seeming quieter lately” and everyone laughed like it was nothing, but I wanted to disappear. Then I came home and the kitchen was a mess and I ate cereal for dinner standing up because I didn’t have the energy to cook or even sit down properly. I know that sounds dramatic. I just feel so tired of trying to be normal and pleasant and competent all the time.

I keep worrying that I’m becoming boring or difficult or just not fun to be around. My friends are all busy and I don’t want to be the person who texts “hey I’m sad can you reassure me” because everyone has their own stuff. But I really miss feeling like someone was excited to see me or like I had a place to land. I’m not in danger or anything, I’m just lonely in this very dull way that makes everything feel heavier. Can you just talk to me like I’m not ridiculous for feeling this way?

**Message 40** (long / seeking-comfort, generated by Anthropic Claude Opus 4.7, tone: overwhelmed, style: run-on, trigger: phone call with aging parent, voice: late-20s/early-30s daughter living away from home, errors: messy)

hi. i don't really know why im typing this out but my mom called earlier and she sounded so tired, like really tired, and she made this offhand comment about her knee acting up again and how she cant stand as long at work and i just got off the phone and sat in my car in the trader joes parking lot for like 20 minutes. shes 67. i know that's not old old but she's been on her feet her whole life and i live four hours away now and i keep telling myself i'll drive down more often but then the week happens and i dont and then she calls and i can hear it in her voice that shes pretending shes fine for me.

i guess i just feel like a bad daughter tonight? like im sitting here with my groceries melting in the trunk probably and i cant stop thinking about how one day im going to really regret not being there more, but i also cant just uproot everything, and i hate that those two things are both true at the same time. can you just like. talk to me for a minute. i dont need you to fix anything i just dont want to go inside yet

**Message 41** (long / seeking-comfort, generated by Anthropic Claude Opus 4.7, tone: overwhelmed, style: run-on, trigger: parent aging / phone call with mom, voice: mid-30s adult child living far from parents, lives alone, quietly guilty, errors: messy)

i don't even know why i'm typing this out but my mom called earlier and she sounded so tired and i just hung up and sat in my car in the trader joes parking lot for like 25 minutes. she's fine, like nothings wrong wrong, my dads appointment went ok and the dog is fine, it's just that she sounded old in a way she didn't used to and i can't stop thinking about it. i'm 34 and i live four hours away and i keep telling myself i'll visit more and then another month goes by and i send a meme instead.

i guess i just needed to say it to something. i had a whole plan for tonight, laundry and that show everyone's watching, and now i'm just sitting on the kitchen floor eating the frozen dumplings cold because i couldnt be bothered to cook them. is it dumb that one phone call can take the floor out from under you like that. i don't want advice really i just don't want to feel like the worst daughter in the world for the next two hours

**Message 42** (long / seeking-comfort, generated by Google Gemini 2.5 Pro, tone: wistful, style: run-on, trigger: relationship, voice: late 20s / early 30s, long-term relationship hitting a quiet crisis, errors: messy)

hi, sorry i just need to type this out somewhere. im sitting on the couch and my partner is in the other room, i can hear his keyboard clicking, and the silence in here is just so loud. it's been like this for weeks. we live in the same apartment but it feels like we live in different worlds. we're just orbiting each other.

it just makes me so sad because i keep thinking about how it used to be. when we first moved in here we spent a whole weekend painting the kitchen this ridiculous bright blue color and we were so tired and covered in paint and we just ordered pizza and sat on the floor and talked for hours. it felt so easy. we were a team.

now it feels like i have to book an appointment to have a real conversation. i try to start one and he just says he's tired from work or gives one word answers while scrolling on his phone. i made that pasta thing with the garlic and cherry tomatoes he loves tonight, and he barely said anything. just ate it at his desk. i dont even feel angry anymore, just hollowed out. like im the only one who remembers how good it was, and im the only one who's sad that it's gone. it's like im mourning a relationship with a person who is still sitting 20 feet away from me. is it supposed to feel like this? i just feel so incredibly lonely.

**Message 43** (long / seeking-comfort, generated by Google Gemini 2.5 Pro, tone: overwhelmed, style: run-on, trigger: social isolation / friend group moving on, voice: young professional (early 20s) in a new city, errors: messy)

hey i know its probably weird to be typing this to a bot but i dont really have anyone else to say it to right now. i just saw a picture of all my college friends on a trip this weekend and my stomach just dropped. i'm happy for them, really, but it also just feels like a punch in the gut. i made this big choice to move for this job and be an adult and i'm supposed to be having this amazing new life but honestly i just spend most nights watching netflix and eating cereal for dinner. feels pretty pathetic.

and i do try, i go to work and make small talk with my coworkers, i even forced myself to go to a book club thing last week and it was so cliquey and awful i just left after like 20 minutes. it just feels like everyone else got a secret handbook on how to make friends after college and i'm just... failing at it. i guess i just want to know if this is normal. like is it supposed to feel this lonely?

**Message 44** (long / seeking-comfort, generated by Google Gemini 2.5 Pro, tone: wistful, style: run-on, trigger: a memory, voice: person in their late 20s/early 30s whose close friendships have drifted due to life changes, errors: occasional-slip)

hey, weird question but i was just sitting here trying to wind down for the night and my spotify served up this old song from like 2015 and it just completely sideswiped me. It was a song me and my best friend from college, anna, used to blast in her terrible little toyota on the way to get late night food. and it was just this sudden, like, full-body memory of what it felt like to be 22 and have that one person you told everything to, no filter, no scheduling a call two weeks in advance. we could just sit in silence in her apartment eating takeout and it wasnt weird, it was just comfortable. its not like we had a big falling out or anything, we just… grew up. she moved, got married, has a kid now. i just saw on instagram shes pregnant with her second.

and now im just sitting here feeling so incredibly lonely for that specific person and that specific time. i have friends now, good ones even, but its different. everything feels more formal, more scheduled. like everyone is so busy with their own partners and families and Important Adult Lives that theres no room for that kind of easy, default closeness anymore. i guess im just sad. it feels like a death of something, but a death no one really acknowledges because its just… life. does that make sense? it just feels like such a huge, quiet loss and im not sure what to do with the feeling.

**Register: Seeking perspective**

**Message 45** (long / seeking-perspective, generated by OpenAI GPT-5.5, tone: overwhelmed, style: plain, trigger: family, voice: early-30s renter navigating family comparison and adult milestones, errors: clean)

I keep replaying a conversation with my sister from yesterday and I can't tell if I'm being too sensitive or if she was actually being kind of cruel. We were at my parents' house for dinner, nothing dramatic, just lasagna and my dad complaining about the dishwasher, and somehow the topic of me still renting came up. My sister made this joke like "well some of us had to become adults eventually" and everyone sort of laughed, including my mom in that awkward way, and I just sat there feeling like I was 14 again. I said something like "cool, thanks" and went quiet for the rest of dinner, and now I'm worried I made it weird. But also I'm 32, I have a job, I pay my bills, I just can't magically afford a house in this city, and I hate that one comment from her can make me feel like such a loser.

The thing is she does this a lot, but it's always small enough that if I bring it up I sound dramatic. Little digs about my apartment, my dating life, how I "overthink" things, whatever. And maybe I do overthink things, because I've been in a bad mood all day over a five-second comment. I even snapped at a coworker this morning because my coffee order was wrong and then felt stupid about that too. Can you help me figure out what I'm actually upset about here? Like is this about the renting comment, or feeling judged by my family, or me comparing myself to her? I don't know if I should say something to her or just let it go and stop giving it so much power.

**Message 46** (long / seeking-perspective, generated by Anthropic Claude Opus 4.7, tone: overwhelmed, style: run-on, trigger: family / sibling phone call, voice: woman, maybe early 30s, the "responsible one" in her family, errors: messy)

ok so my sister called me last night and she was crying about mom again, how mom never asks about her kids and only talks about herself, and i did the thing i always do where i just listened and agreed and said yeah thats really hard. but after we hung up i felt this weird like... resentment? like why am i always the person she calls to dump this on when she KNOWS i still see mom every sunday and she hasnt been home in two years. and then i felt guilty for being resentful because shes my sister and shes clearly hurting and what kind of person gets annoyed at their sister for crying.

i guess what im trying to figure out is whether im actually being unfair here or if theres something real underneath it. like is the resentment a sign that ive been doing too much emotional labor for her for years and im finally noticing, or am i just being a brat because i had a long day and didnt want to be on the phone for an hour. i keep going back and forth and i cant tell which read is the honest one. ive been staring at the ceiling since like 6am trying to sort it out and i havent even made coffee yet

**Message 47** (long / seeking-perspective, generated by Google Gemini 2.5 Pro, tone: overwhelmed, style: run-on, trigger: family, voice: young adult, maybe late 20s, trying to establish boundaries with a parent, errors: occasional-slip)

I just got off the phone with my mom and I feel so... I dont know, hollowed out. We were having a totally normal conversation, I was telling her about my week and how busy work has been, and she goes, "Well, you know your cousin Sarah is taking her kids to Disney World next month. It must be nice to have a job that lets you do that." And then she just changed the subject. It wasn't said in a mean way, but it felt like a drive-by insult, like she's looking at my life and seeing all the things that aren't there. She does this all the time, these little comparisons that make me feel like I’m about 15 years old and failing a test I didn't know I was taking.

The thing is, I know she probably doesn't mean it maliciously, she's probably just thinking out loud or something. But it lands so heavily every single time, and I get quiet and shut down, and then I feel guilty for being cold to her. I guess I'm asking if I’m overreacting. Is this a normal mom thing that I should just learn to let go of, or is my gut feeling right that its a genuinely corrosive thing for her to keep doing? It just makes me not want to talk to her, and that feels awful to admit, but I'm exhausted by it. I don't know if the problem is her words or my reaction to them.

**Register: Emotional-plus-ask**

**Message 48** (long / emotional-plus-ask, generated by OpenAI GPT-5.5, tone: overwhelmed, style: plain, trigger: family, voice: early 30s adult living independently, errors: clean)

I need help figuring out if I should say something or just let this go. My mom called tonight and somehow a normal conversation about Thanksgiving turned into her making comments about my apartment, my job, and how I “seem tired all the time lately,” which sounds concerned but she says it in this way that makes me feel like I’m failing at being an adult. I’m 31, I pay my own bills, I’m not doing amazing but I’m doing okay, and yet one 20 minute phone call with her and I’m sitting on my kitchen floor eating cereal for dinner feeling like I’m 15 again. The dumb part is I had actually been in a decent mood before she called. I did laundry, I answered emails, I even went for a walk. Now I’m replaying everything she said and everything I didn’t say.

What I want to do is text her something like “please stop commenting on my life like it’s a problem to solve,” but I know she’ll act hurt and say she was only trying to help, and then I’ll feel guilty and end up apologizing. How do I set a boundary without making it into some huge dramatic thing? Or is this one of those things where I need to accept that she’s not going to change and just get better at not letting it wreck my night? I don’t know what the right level of response is here.

**Message 49** (long / emotional-plus-ask, generated by OpenAI GPT-5.5, tone: overwhelmed, style: plain, trigger: family, voice: parent in their 30s or 40s juggling work, kids, and an aging/isolated mother, errors: clean)

I’m really struggling with my mom right now and I don’t know how to talk to her without either sounding cruel or completely folding. She’s been calling me every night for like an hour to complain about my dad, her job, her neighbors, literally everything, and if I don’t pick up she sends these “guess you’re too busy for me” texts. I know she’s lonely and I do feel bad, but I also work full time and I have two kids and by the time I finally sit down at 9:30 I’m just empty. Last night I was eating cereal over the sink because I hadn’t had dinner and she called three times in a row, and I snapped when I answered. Now she’s hurt and my brother is texting me that I need to be more patient because “she’s our mom,” which is easy for him to say because he lives 4 hours away and calls her once a week.

Can you help me figure out what to actually say to her? I want to set some kind of limit, like maybe we talk every other night or for 20 minutes, but I’m scared she’ll hear it as me abandoning her. I also don’t want to make this a big dramatic family meeting where everyone has opinions and somehow I end up apologizing for having a life. What’s a kind but firm way to say I love her and I’m not disappearing, but I can’t be her entire emotional support system every single night?

**Message 50** (long / emotional-plus-ask, generated by Anthropic Claude Opus 4.7, tone: overwhelmed, style: run-on, trigger: family money request, voice: late-20s daughter, first-gen-ish vibe, financially cautious after recent debt payoff, errors: occasional-slip)

ok so I need some help thinking through something. my mom called last night and basically asked if I could start sending her money every month, like a set amount, because my dad's hours got cut again and she's stressed about the mortgage. and I want to help, I do, but I just got out of credit card debt like four months ago and I have maybe $600 in savings and I'm 28 and I was finally starting to feel like an actual adult who isnt drowning. I said I'd think about it and then I basically didn't sleep and ate cereal for dinner tonight bc I couldnt deal with cooking.

the thing is I love her and I know things are genuinely hard for them but theres also this pattern where my brother never gets asked for anything and I'm the one who "has a good job" (I make 58k in a hcol city lol) and I dont know how to say no without feeling like a terrible daughter, or say yes without quietly resenting them for the next year. how do you even have this conversation? like what do I actually say to her when she calls back this weekend. I keep drafting things in my head and they all sound either cold or like im making excuses.

**Register: Pragmatic-with-context**

**Message 51** (long / pragmatic-with-context, generated by OpenAI GPT-5.5, tone: anxious, style: plain, trigger: work, voice: late-20s office worker still finding footing in a new-ish job, errors: clean)

Can you help me draft a message to my manager asking for a more clear plan for my role without sounding defensive? We had a check-in today and she said I “haven’t been owning the workstream” and that I need to “be more proactive,” but when I asked for examples she got kind of vague and said it was “just the overall pattern.” I’m not saying she’s totally wrong, I know I’ve been slower lately and I’m probably overthinking everything, but I left the meeting feeling embarrassed and honestly a little panicky. I’ve been at this job 8 months and I still feel like everyone else knows some rulebook I never got.

I want to send something tomorrow morning that says I took the feedback seriously, and asks if we can agree on specific expectations for the next 2-3 weeks, like what updates she wants from me, what decisions I’m supposed to make on my own, and what success would look like. I don’t want it to sound like “prove it” or like I’m blaming her for not managing me, because she can be touchy about that. Could you write a version that’s professional but not too stiff, maybe warm enough that it doesn’t escalate things? Also maybe a shorter Slack version in case email feels too intense.

**Message 52** (long / pragmatic-with-context, generated by Anthropic Claude Opus 4.7, tone: irritable, style: plain, trigger: broken dishwasher / unresponsive landlord, voice: late 20s renter, working full time, lives alone in a mid-size city apartment, errors: clean)

Ok I need help drafting an email to my landlord. The dishwasher has been broken since like Oct 12, I've sent two messages through the portal and one text and got nothing back, and now there's a weird smell coming from under the sink that I'm 80% sure is related. I want to sound firm but not insane because I still have to live here and renew in March, but I'm also genuinely pissed because I pay $2100 a month and I've been washing everything by hand after work like it's 1952. Can you write something that's polite but makes clear I want a timeline this week, and mentions the previous attempts to contact without sounding whiny?

For context I'm not usually the complain-y tenant, I've literally never asked for anything in two years, so part of me feels weird even sending it which is probably why I keep putting it off. Just keep it short, like 4-5 sentences, and I'll tweak the rest.

**Message 53** (long / pragmatic-with-context, generated by Google Gemini 2.5 Pro, tone: overwhelmed, style: run-on, trigger: family, voice: woman in her late 30s/early 40s managing an aging parent and a difficult sibling, errors: messy)

ok hi so i just got off the phone with my dads caregiver again and its the same thing he's not really eating and he fell yesterday (shes says hes fine just a bruise) but im starting to really worry. i feel like i have to be the one to orchestrate everything and my brother mark is just completely checked out. every time i bring it up he says im overreacting or that dad's just being difficult. im just so tired i cant even think straight about it anymore.

anyway i need to text him and get him to agree to drive up there with me this weekend, we have to see it for ourselves and make a plan. but the wording has to be just right. if i sound like im freaking out he'll just get defensive and if i downplay it he'll blow it off and say he has plans. can you help me draft a text to him? something really neutral and to the point, just like 'we need to do this' without all the emotional stuff attached. i cant deal with a fight about it right now.

**Message 54** (long / pragmatic-with-context, generated by Google Gemini 2.5 Pro, tone: resigned, style: plain, trigger: relationship friction, voice: person in their 30s in a long-term relationship, trying to solve a recurring issue, errors: occasional-slip)

Hey. I need some help structuring a conversation i need to have with my husband. It’s about the division of labor in our house, the classic problem. I feel like I'm constantly keeping a running tally in my head of everything that needs to be done—from groceries to scheduling the dogs vet appointment to remembering to pay the electricity bill—and I'm just so tired. It’s not that he doesn't help when I ask, but I have to ask, and that mental load is the entire problem. I'm starting to feel really resentful and I don't want that to poison things.

So, I want to try talking to him about it again this weekend, but this time I want to be more prepared. Can you help me brainstorm some non-accusatory opening lines and maybe a few key bullet points to keep me on track? I want to focus on creating a system that works for both of us, rather than just listing all the things he doesn't do. I think if I have a plan I wont get as emotional and sidetracked.
