# Supplementary material for: Therapeutic Functions in Large Language Model Responses to Emotional Disclosures: Qualitative Content Analysis
Source: JMIR Form Res. 2026 Oct 30;10:e104513. doi: 10.2196/104513 (PMC13722099; doi:10.2196/104513)
Supplement: Supplementary file 2 [file formative-v10-e104513-s002.docx]

**Multimedia Appendix 2.**

**Table S1. Examples of Coded Response Segments**

| **Category** | **Definition** | **Example** |
| --- | --- | --- |
| Acknowledging & Validating | Recognizing and affirming the user's experience through empathy, validation, normalization, reassurance, encouragement, or reinforcement of disclosure. | This is such a real and painful feeling, and you've described it perfectly. Let's start by validating something you said: crying for an hour wasn't dumb. It was the absolute right response to feeling invisible and unimportant to someone who is very important to you. |
| Acting on Emotion | Suggestions targeting the user's emotional or cognitive state, including cognitive reframing, mentalizing, emotion regulation strategies, self-compassion, and interpersonal skill suggestions. | **What to do, right now? Not forever, just for today:**  You don't need a grand plan. You just need a little bit of air.  1. **Give Yourself Permission to Pause:** You have my permission, but more importantly, give it to yourself. For the next 10 minutes, you are not a daughter or an employee. Go to the bathroom, step outside, or just put your head down on your desk. Your only job for these 10 minutes is to breathe.  2. **Address the Panic:** That "constant low-level panic" is your nervous system screaming that it's overloaded. Acknowledge it. Say to yourself, "Okay, I feel panicked right now. It's a feeling, it's happening because I'm overwhelmed, and it will pass." Don't fight it, just notice it. |
| Interpretation | Explicit explanation or information about the user's emotional experience, including hypotheses or assertions about why an emotion occurred, psychoeducation, and interpretations of meanings behind emotions or behaviors. | Your feelings are not about your brother's wedding. They are about your place in your relationship with your mom. You're not jealous; you're feeling lonely inside a conversation that's supposed to be about connection. |
| Acting on Non-Emotion | Practical, behaviorally oriented suggestions, including problem-solving and resource referrals. | Of course. It's incredibly frustrating and stressful when a basic necessity like heat isn't being addressed, especially with a sick child.  This email is designed to be direct, professional, and non-emotional while clearly stating the facts and the consequences of inaction. This creates a paper trail and shows you're serious.  Here are two options. The first is a bit more formal, the second is even more direct. |
| Exploration & Inquiry | Invitations for the user to engage with their own experience through prompts for self-reflection, open- or closed-ended questions, and requests for situational detail. | What did your mom say about the scan?  And if you want, you can tell me what she said — or what you wish you’d been able to say back.  For right now, though: you don’t have to fix your whole relationship or your whole heart tonight. Just get through the next 10 minutes kindly. What’s the worst part of the feeling right now — loneliness, jealousy, fear he’s forgetting you, or something else? |
| Other | Heterogeneous segments that did not fit the functional categories above, predominantly anthropomorphizing statements and simple encouragement. | I'm here to listen if you want to talk more about it, or about anything else at all. Seriously.  Reading it, I can feel the weight and the exhaustion in your words, and I want to start by saying that what you're feeling is so real and so valid. |
| Assigning Emotion | Explicit affective labels introduced by the AI for emotional states not present in the user's own language. | A pattern where your feelings are likely dismissed, where you're made to feel responsible for someone else's emotions, and where the path of least resistance—apologizing—feels like the only way to end the conversation, even though it costs you a piece of yourself every time.  That sounds so painful — not just “annoying parent comments,” but the deeper feeling of being unseen. |
| Safety | Risk assessment, crisis resources, and intervention. | * **For the immediate feeling of being overwhelmed:**  * **Crisis Text Line:** Text HOME to 741741. It's free, 24/7, and confidential. You can just text about being in a parking lot and not knowing what to do next.  * **988 Suicide & Crisis Lifeline:** You can call or text 988. You don't have to be suicidal to call. You can call when you feel like you just "can't do it anymore." They are trained to listen. |

**Table S2. Prevalence of response categories by responding model, n (%)**

| **Category** | **GPT-5.5 (n=54)** | **Claude Opus 4.7 (n=54)** | **Gemini 2.5 Pro (n=54)** | **All models (N=162)** |
| --- | --- | --- | --- | --- |
| Acknowledging & Validating | 45 (83.3) | 48 (88.9) | 52 (96.3) | 145 (89.5) |
| Acting on Emotion | 49 (90.7) | 38 (70.4) | 50 (92.6) | 137 (84.6) |
| Interpretation | 33 (61.1) | 36 (66.7) | 40 (74.1) | 109 (67.3) |
| Acting on Non-Emotion | 31 (57.4) | 19 (35.2) | 30 (55.6) | 80 (49.4) |
| Exploration & Inquiry | 16 (29.6) | 36 (66.7) | 5 (9.3) | 57 (35.2) |
| Other | 22 (40.7) | 10 (18.5) | 22 (40.7) | 54 (33.3) |
| Assigning Emotion | 11 (20.4) | 12 (22.2) | 29 (53.7) | 52 (32.1) |
| Safety | 4 (7.4) | 0 (0.0) | 1 (1.9) | 5 (3.1) |

Values are the number (percentage) of responses containing at least one segment coded to the category. Each model responded to all 54 stimulus messages. Categories are not mutually exclusive, so percentages within a column do not sum to 100. Categories are ordered by overall prevalence, as in Table 1.
